# Hydroforming of Toroidal Bellows: Process Simulation and Quality Control

**DOI:** 10.3390/ma14010142

**Published:** 2020-12-31

**Authors:** Mengsi Ye, Huifang Li, Yougang Wang, Caifu Qian

**Affiliations:** 1 Institute of Mechanical and Electrical Engineering, Beijing University of Chemical Technology, Beijing 100029, China; 15624964872@163.com; 2 Dalian Yiduo Pipeline Limited Company, Dalian 116318, China; wyg2357@126.com

**Keywords:** toroidal bellows, hydroforming, finite element simulation, roundness of wave-shape, wall thickness reduction

## Abstract

Having higher capacity to undertake pressures and larger compensation ability compared with the U-shape bellows, toroidal or Ω-shape bellows are being more and more widely used in engineering. The wave-shape and wall thickness reduction of bellows are the most important parameters for measuring the hydroforming quality of the bellows. In order to provide references for actual manufacturing, it is valuable to study the factors influencing the hydroforming process and quality of the bellows. In this paper, finite element simulations of the hydroforming process of a monolayer and single-wave toroidal bellows and a two-layer and four-wave toroidal bellows were carried out. Stress and strain distributions before and after unloading were analyzed and the wave height and wall thickness reduction were examined. The numerical results were verified by the actual hydroforming measurements. In addition, ranges of the significant structural or operating factors for producing better bellows were studied and a formula to compute the wall thickness reduction was fitted based on the sufficient numerical results of the hydroforming simulations.

## 1. Introduction

In engineering practice, there are two methods to manufacture bellows, i.e., integral hydroforming or split jointing. The former is more advanced and can be used in the manufacture of the multi-layer multi-wave expansion joints. A lot of studies can be found in the literature on the hydroforming of bellows, especially numerical simulations. In order to analyze the tensile stress and thickness reduction at the wave peak of joint bellows of AISI 316 steel for different values of diameter, Estrems et al. [1] performed finite element simulations and the results are compared to the recommendations given by the EJMA standard. Liu et al. [2] simulated the hydroforming process of a three-layer convoluted bellows with ABAQUS software. Results show that the equivalent stress, strain values and wall thickness reduction are different in different layers with the inner layer having a larger reduction than that in the middle and outer layers. In addition, the wall thickness reduction in the peak is the most serious, and it decreases gradually from wave crest to trough.

There are many factors affecting the quality of bellows hydroforming, including the height of tube blank, pressure load, axial feed and springback, etc. Jooybari et al. [3] studied the effect of pressure path on the quality of hydroforming bellows. The simulation results show that the pressure path affects the production of the desired parts or the occurrence of bursting or wrinkling defects in the products. Faraji et al. [4,5] studied the influence of the mechanical properties, die stroke, internal pressure, and axial feeding on the quality of hydroforming of bellows. Results show that with the increase of Young modulus, the springback of the manufactured bellows decreases. In addition, their study also demonstrated that increasing the diestroke and internal pressure leads to excessive thinning and springback. The effect of the specific manufacturing parameters including forming pressure, punch speed, size ratio on minimum thickness and formability of bellows were analyzed with finite element method by Lin et al. [6]. Results show that the forming parameters have some influences on the minimum thickness and the formability, and the dimension ratio is the most significant. The finite element analysis was applied to analyze the influence of internal pressure and the speeds of the punch and die oncrease by Lee and Kim [7]. The study concluded that it is internal pressures rather than the speeds of the punch and die affected the height of crest and with increasing the internal pressures, the crest is formed more accurately. Qian and Chen [8] presented theoretical calculation formula for the corrugated pipe hydroforming parameters (unabated wave pressure, axial thrust and single wave expansion length), and introduced a practical hydroforming process method. The theoretical formula of force load applicable to the specific bulge coefficient was deduced, and the accuracy of the empirical formula based on this formula was verified by Artemov [9]. Liu et al. [10] studied the springback behaviors of bi-layered non-homogeneous bellows in hydroforming. Results show that the U-shaped convolution profile is changed to tongue shape accompanied by a 2.5–38.5% axial elongation and a 0.1–0.6% radial shrinkage after springback, and the springback tendency grows with the increase of number of layers, the improvement of mechanical properties of material, and the decrease of expansion ratio.

Ubido [11] examined the geometric properties of multi-ply bellows, and then develops a theory based on idealised geometric properties using expected load application methods. Some outstanding problems in bellows application are examined and methods of solution suggested for each problem discussed. Anderson [12] presented a method of evaluating the design application of bellows on the basis of calculated allowable stresses. This method allows evaluation of both deflection stresses and pressure stresses on convoluted bellows, convoluted bellows with reinforcing rings and toroidal bellows. Kazuyuki [13] have developed a simplified theoretical modeling of creep behavior of bellows. The relation between forces and rates of creep displacements has been derived using Norton’s law for creep property of bellows material. The validity of this method has been discussed mainly from the viewpoints of predictability and efficiency through some example analyses. Pavithra [14] investigates the fatigue behaviour of U shaped hydroformed bellows using the finite element method. The fatigue tests were also conducted at room and elevated (650 °C) temperatures. The experimental results of the fatigue test correlate well with the numerical results. Kang et al. [15] used a single-step tube hydroforming process to make prototype tubular bellows with simultaneous control of the internal pressure and the axial feed. The study shows that a single-step hydroforming process can be used to form tubular bellows with various shapes.

In this paper, hydroforming process of a monolayer and single-wave toroidal bellows and a two-layer and four-wave toroidal bellows are simulated using finite element method. Stress distributions and wall thickness reduction of the bellows are investigated. The numerical results are verified by the actual hydroforming measurements. Structural and operating parameters influencing the hydroforming quality of the bellows are studied in order to provide references for actual hydroforming of the toroidal bellows.

## 2. Numerical Simulations

### 2.1. Simulations of Hydroforming Process of a Monolayer and Single-Wave Toroidal Bellows

#### 2.1.1. Geometric and Mesh Model

**Figure 1:**
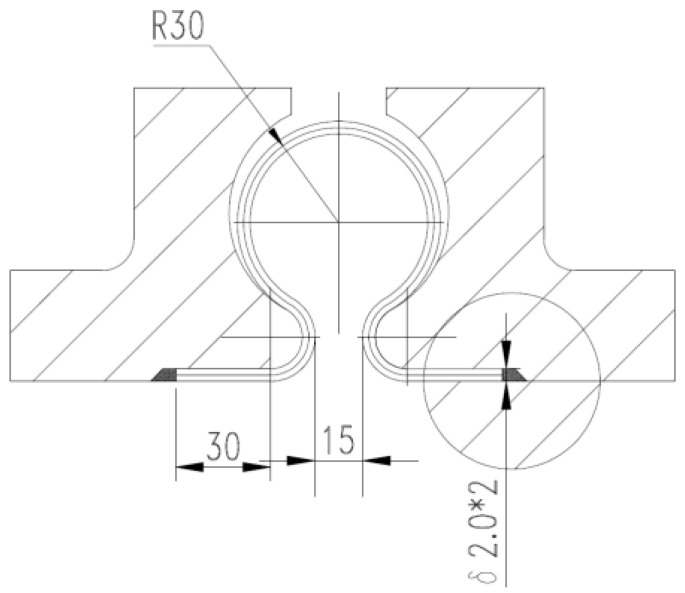
Illustration of a typical toroidal bellows (mm).

Figure 1 shows a typical toroidal bellows structure which was provided by Dalian Yiduo Pipeline Limited Company (Dalian, China). In this study, the finite element software, ANSYS-Workbench is used to simulate the hydroforming process of the bellows. As the simulation involves geometric nonlinear, material nonlinear and contact nonlinear, reasonable simplifications were made for the simulation in order to reduce the calculation time. As the geometry of the mold and pipe blank is axisymmetric, and the forces applied are also axisymmetric during the hydroforming process, a two-dimensional axisymmetric model is established. The eight nodes element Plane183 which has the property of plasticity, stress hardening, large deformation and large strain is chosen to simulate the hydroforming process.

**Figure 2:**
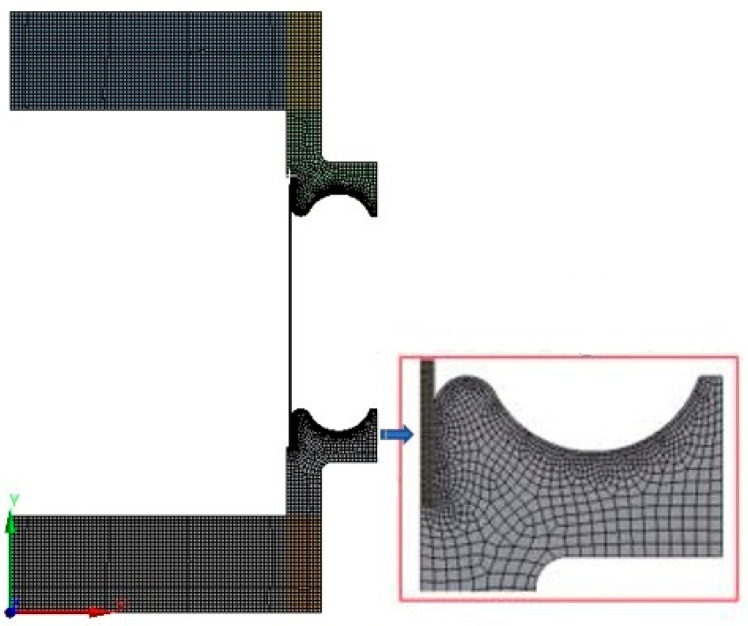
Mesh model of bellows for hydroforming process simulation.

The finite element geometric model of the bellows is composed of the tube blank, stiffening rings, upper and lower molds. For well reflecting the bending deformation of the tube blank during the forming process, three layers of grids are divided along the wall thickness. The grid size is studied to ensure the results independence and final length of the grid size is set to be about 1mm. The mesh model of the bellows for hydroforming is shown in Figure 2.

#### 2.1.2. Material Model

**Figure 3:**
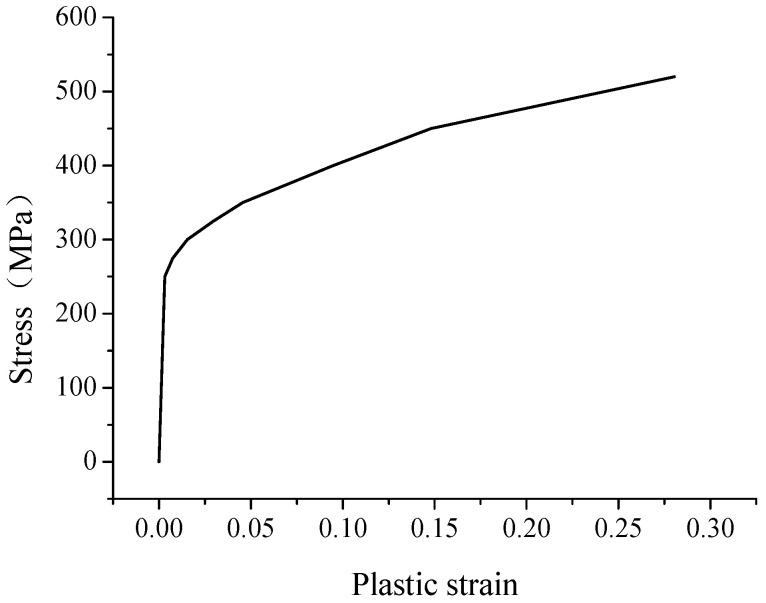
True stress-strain curve of the tube blank material.

It is essential to apply the correct constitutive relationship for the material in the hydroforming process simulation as the large deformation with permanent plastic strain is involved. The material for the bellows is austenitic stainless steel S30403. In this study, true stress-strain curve of the material is applied as shown in Figure 3. There are two hardening criteria in the ANSYS program, namely kinematic hardening and isotropic hardening. In this study, multi-linear isotropic hardening is adopted [16].

Compared with the tube blanks, the rigidity of the material of the top and bottom molds is large, and the deformation should be very small during hydroforming process. Therefore in this simulation, deformation of the molds is neglected or in other words, the molds are set to be rigid bodies with its elastic modulus being assumed to be 10 times of that of the ordinary carbon steel.

#### 2.1.3. Constraint Conditions

**Figure 4:**
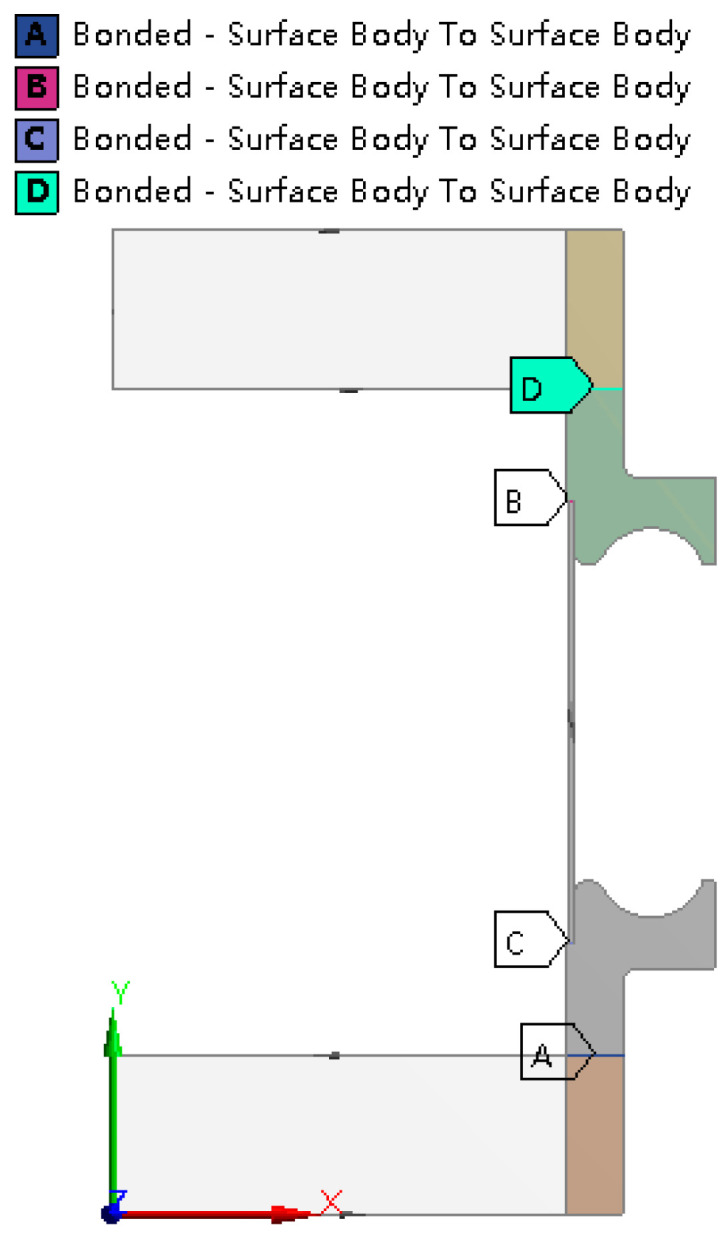
Illustration of bonded contact positions.

**Figure 5:**
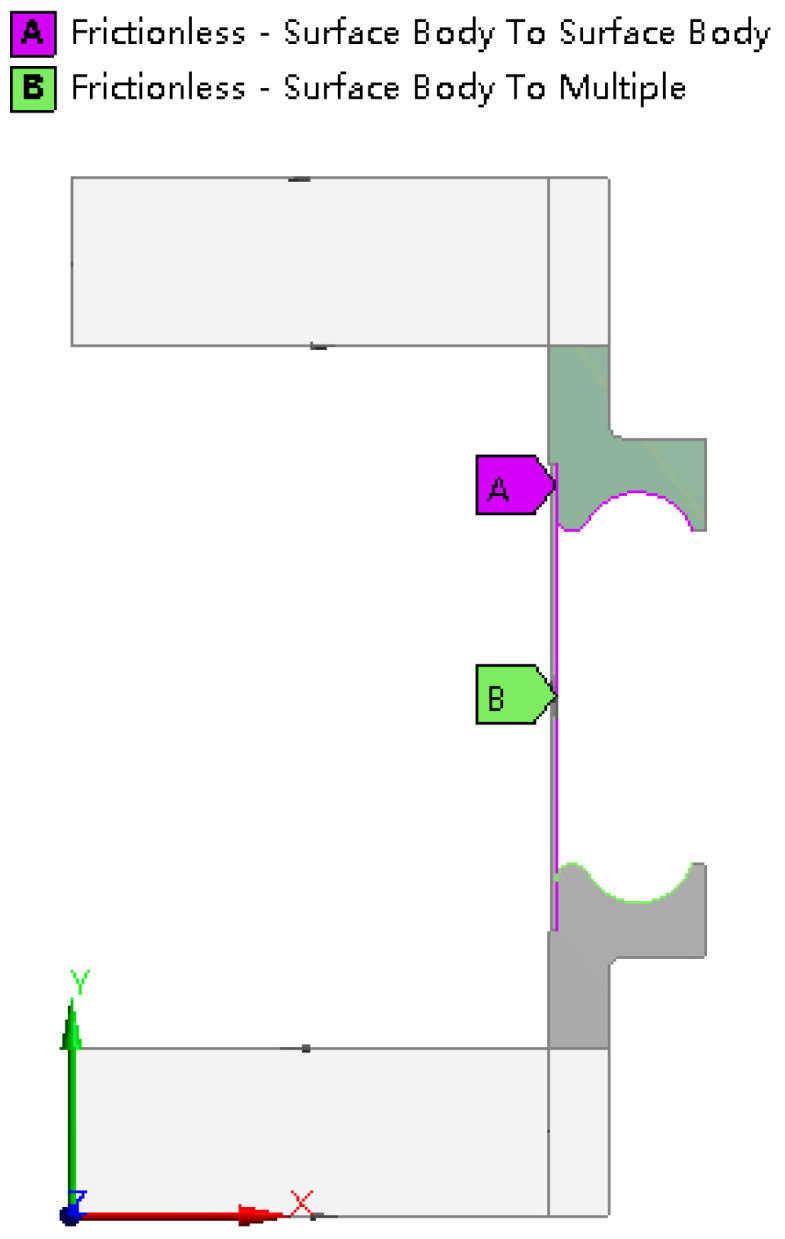
Illustration of frictionless contact positions.

As involving strong plastic deformation, there are a lot of body surface contacts during the hydroforming process of bellows. In the simulation, surface contacts are setting following this principle. If two surfaces are always contacted without sliding and changing in area, the contact is simulated as the bonded contact. Otherwise the contact is considered to be frictional or frictionless contact. For the frictional contact, Coulomb friction law is applied. In this study, the end stiffening rings are always in contact with the molds, so they are set as bonded contacts. Similarly, the ends of the tube blank and the end stiffening rings are also set to be bonded contact, as shown in Figure 4. At the initial stage of forming, the tube blank and the stiffening rings are partly contacted. As the forming process keeps going, the tube is completely contacted with the small arc of the stiffening rings, and partly contacts with the big arc. So during the forming process, the outer surface of tube blank and the middle stiffening rings may be separated or at tangential slip. It turns out after computation that the friction between the contact surfaces have little influence on the hydroforming results, and, thus, frictionless contacts are applied in order to facilitate the convergence, as shown in Figure 5.

**Figure 6:**
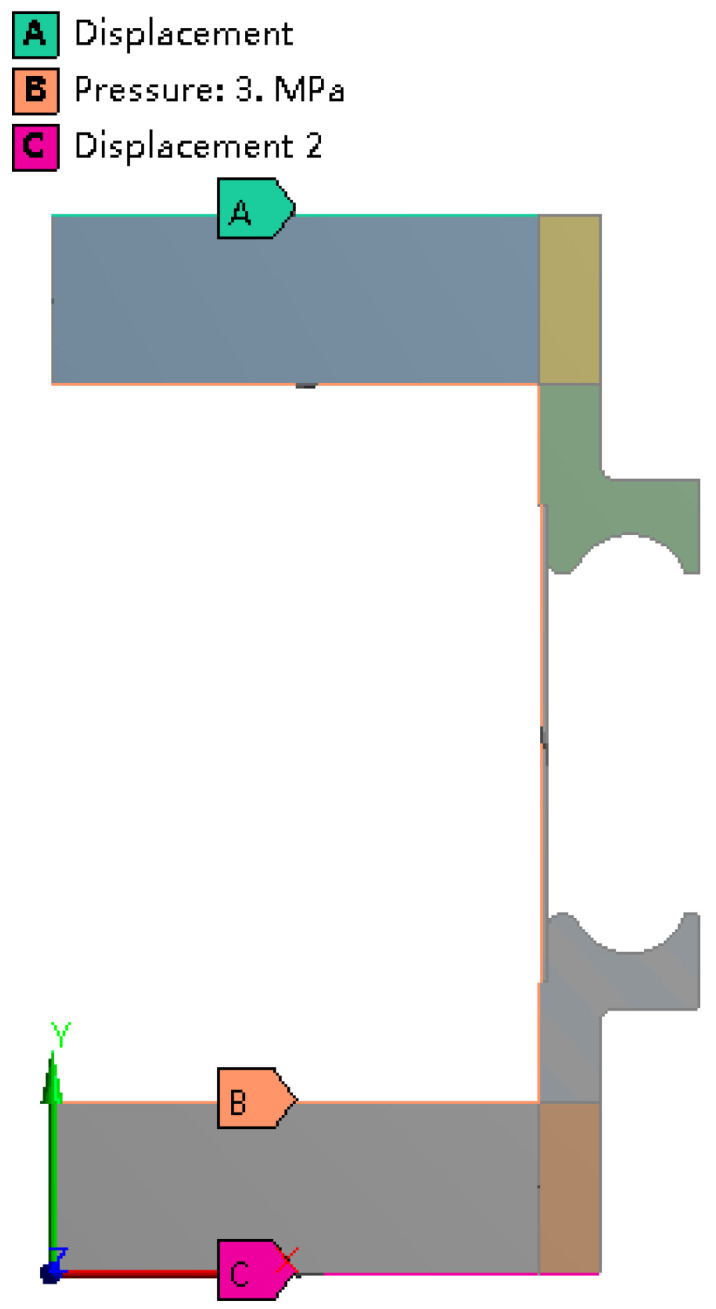
Constraint setting for the simulation.

Hydroforming process of bellows includes three stages, namely bulge stage, fold stage and springback stage. For the toroidal bellows studied here, the pressure increases linearly from 0 to 3 MPa at the bulge stage, then increases linearly to 13 MPa at the fold stage and decreases to 0 at the springback stage. The pressure is loaded inside the tube blank, as indicated by B in Figure 6. At the bulge stage, the stiffening rings are supported by blocks, and the displacement is 0. Specified displacement is loaded on the top mold, as indicated by A in Figure 6. At all stages, the bottom mold has no displacement in any direction, so the displacement is set to be 0 as indicated by C in Figure 6.

#### 2.1.4. Result and Discussion

- Stress distribution

**Figure 7:**
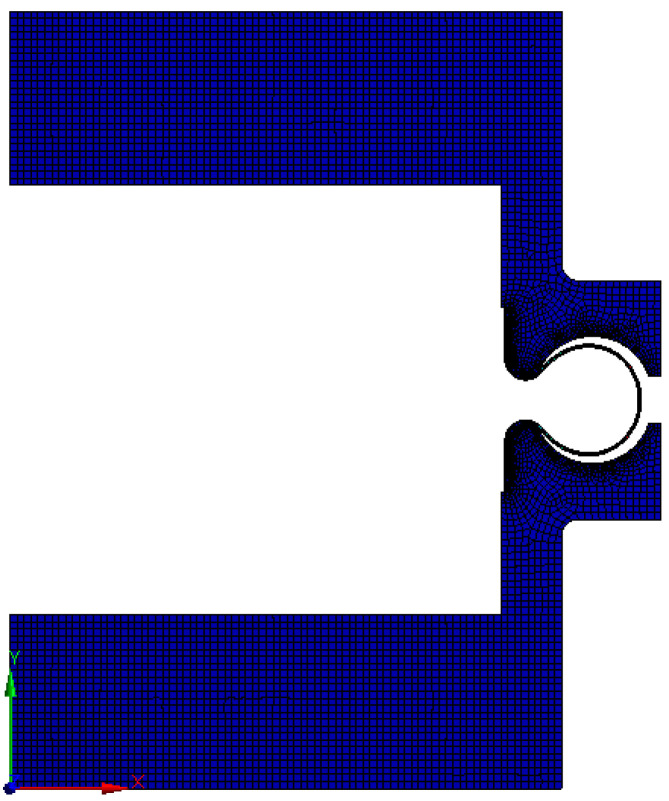
Shape of the bellows after hydroforming.

**Figure 8:**
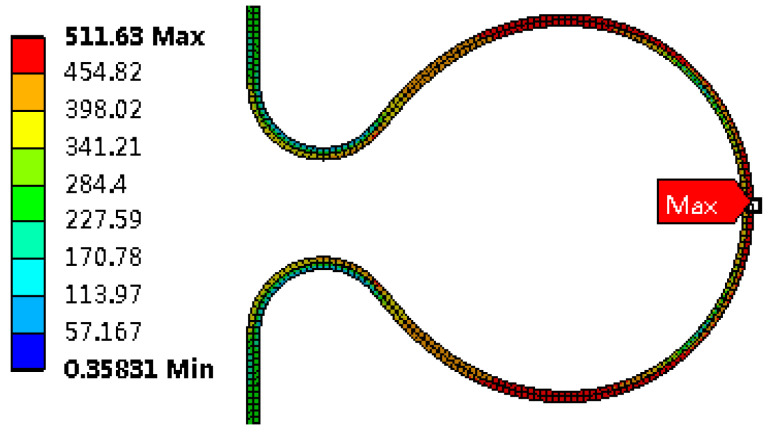
Von Mises equivalent stress distribution before unloading (MPa).

**Figure 9:**
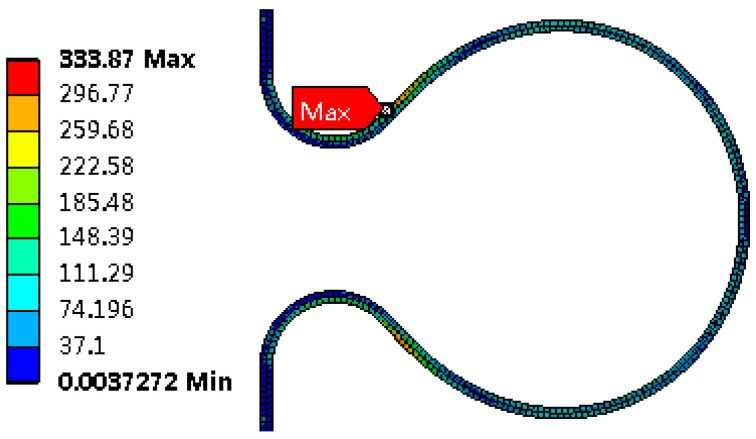
Von Mises equivalent stress distribution after unloading (MPa).

The shape of the bellows after hydroforming is shown in Figure 7. von Mises equivalent stress distribution at the wave before unloading is showed in Figure 8. The maximum equivalent stress is found at the large arc. Due to the constraint of stiffening rings, the von Mises equivalent stress after unloading is largely located in the transition of large arc and small arc and is reduced by about 30% compared with that before unloading, as shown in Figure 9.

It is noted that what Figure 9 showed is the residual stress. Relief of the residual stress may cause structural deformation. So a manufacturer of bellows should pay sufficient attention to the magnitude of the residual stress left in the bellows after hydroforming.

- Strain distribution

**Figure 10:**
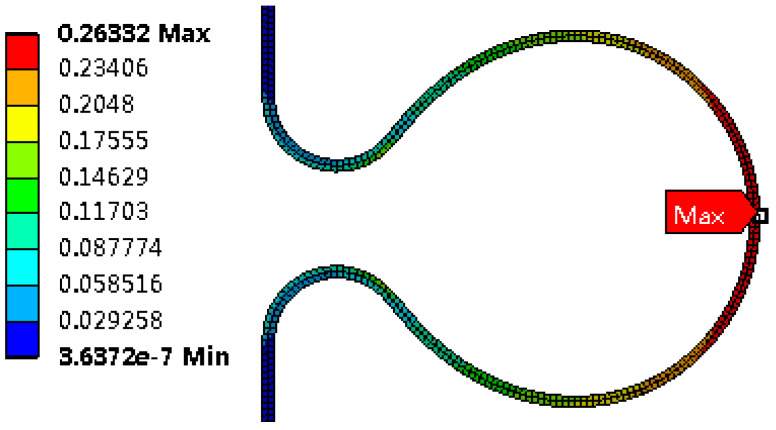
Von Mises strain at the wave before unloading.

**Figure 11:**
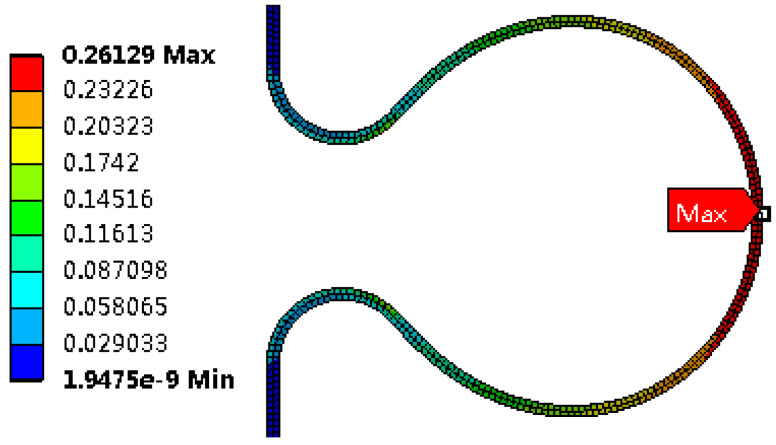
Von Mises strain at the wave after unloading.

After the bellows is formed, the top area of the large arc has the big von Mises equivalent strain. The equivalent strain after unloading is reduced by 0.78% compared with that before unloading, as shown in Figure 10 and Figure 11, indicating that plastic deformation is the main strain at the bellows during the hydroforming.

- Structural parameters of the bellows

The displacements in wave height direction before and after unloading of the toroidal bellows are listed in Table 1. Comparing the displacements in the wave height direction of the tube blank before and after unloading reflects the amount of the elastic recovery or springback after unloading. For example, 77.821 mm of the displacement at the outer wall before unloading substracting 77.263 mm of that after unloading yields the elastic recovery of 0.558 mm. The difference of displacements at the wave peak between the inner wall and the outer wall is the thickness reduction. For example, 77.613 mm of the displacement at the inner wall after unloading subtracting 77.263 mm of that at the outer wall after unloading gives 0.35 mm of the wall thickness reduction at the wave peak of that tube. The relative wall thickness reduction of bellows is 17.5%.

### 2.2. Numerical Simulations of Hydroforming Process of a Multi-Layered and Multi-Wave Toroidal Bellows

**Figure 12:**
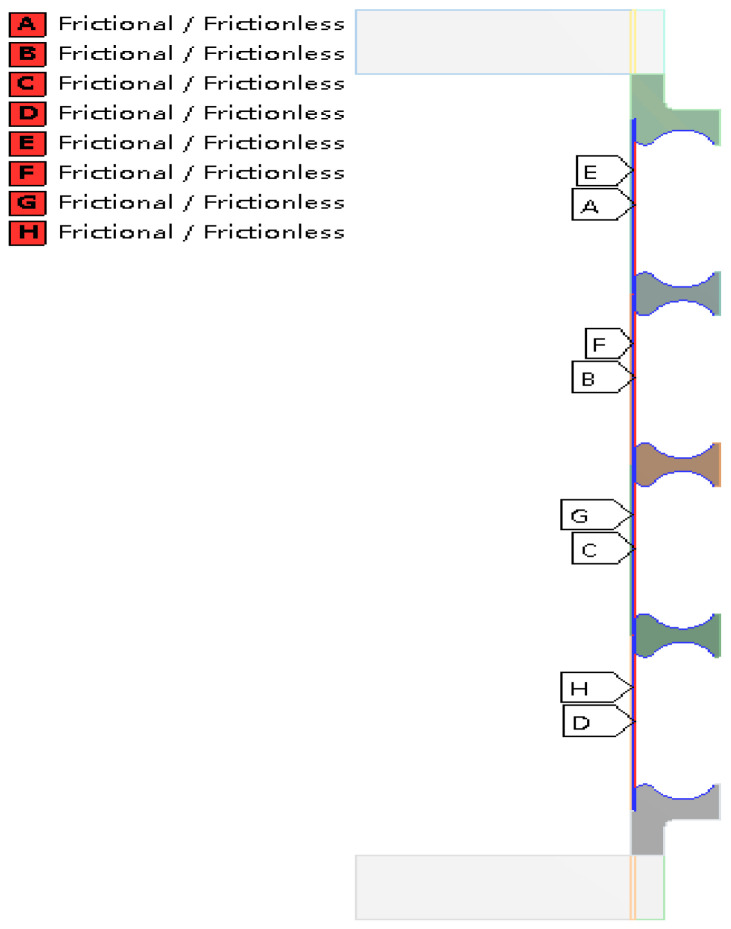
Illustration of contact positions of the two-layers and multi-waved toroidal bellows.

Multi-layer and multi-wave bellows are widely used in engineering. In order to provide references for the hydroforming of these bellows. The hydroforming process of a two-layer and four-wave toroidal bellows was also simulated in this section. The dimensional and material parameters and the constraint conditions of this two-layer and four-wave toroidal bellows are the same as the above monolayer and single-wave bellows. In order to figure out the effect of friction between layer to layer and layers todies, both frictional (friction coefficient is set to be 0.15) and frictionless contacts are applied as shown in Figure 12.

The stress and strain distributions of these two-layer and four-wave toroidal bellows are the same as the monolayer and single-wave bellows and the displacement results in the wave height direction with the frictionless contacts setting before and after unloading of the toroidal bellows are listed in Table 2. The absolute and relative wall thickness reduction of bellows with the frictionless contact setting is listed in Table 3. It is seen that the relative wall thickness reduction at the wave crest is as high as 18%. It is found by comparing the simulations that the difference of the concerned results between frictionless contact setting and frictional contact setting is less than 0.5%.

### 2.3. Practical Measurements

**Figure 13:**
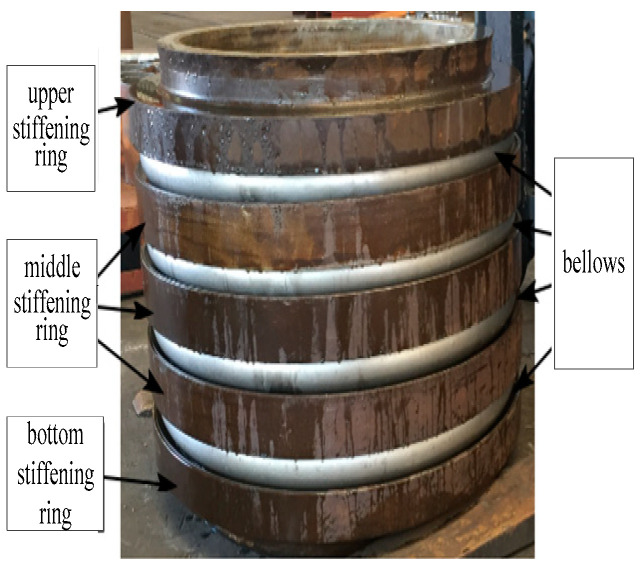
Formed expansion joint.

**Figure 14:**
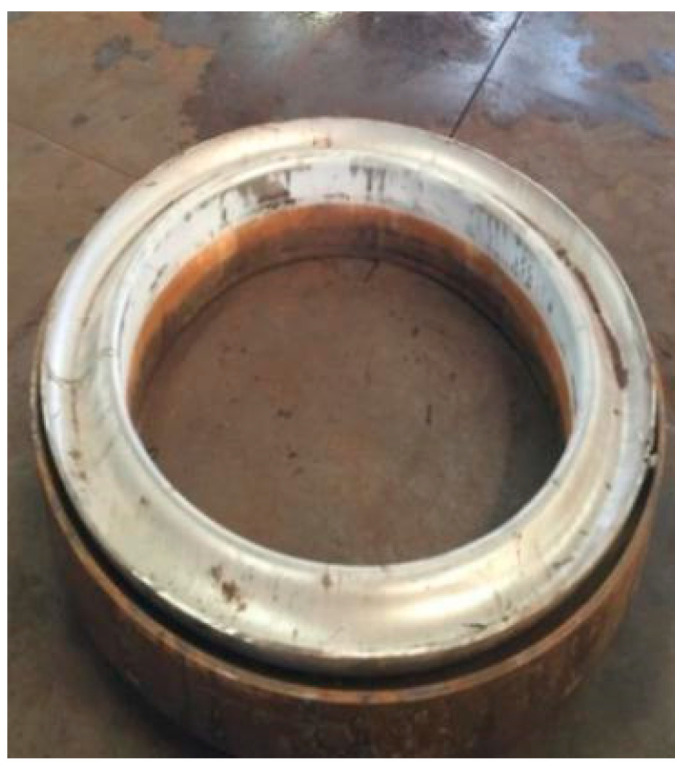
Sliced bellow.

In order to verify the correctness of the simulation results, destructive measurements were carried out on the expansion joint. The formed bellows is shown in Figure 13. After being machined, the sliced wave is shown in Figure 14. Because of the practical restrictions, the wall thickness and the displacement in the wave height direction of the first wave of the sliced bellows are measured by Vernier calipers with the precision of 0.02 mm. The displacements in the wave height direction at the inner wall of the inner tube of other wave were also measured.

Results of comparison between simulations and measurements of the first wave are listed in Table 4 and that of other waves is listed in Table 5. It is seen from the tables that the simulation results and experimental results are in good agreement with the relative errors less than 3.78%. It is implying that the finite element method is feasible and dependable for the numerical simulation of the hydroforming process of the toroidal bellows.

## 3. Quality Control

### 3.1. Requirements of the Structural and Operating Parameters for Hydroforming of Bellows

**Figure 15:**
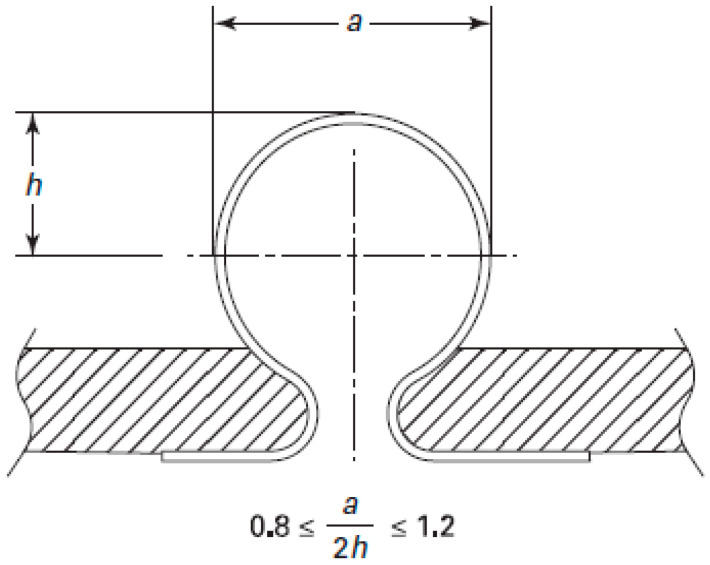
Permissible tolerances for bellows manufacture (mm) [17].

The relative wall thickness reduction (δ) and the ratio of structural parameters (a/2 h) are usually used to reflect the bellows hydroforming quality. Clearly, a better hydroforming quality requires that a/2 h should be close to 1 and δ should be small. According to ASME VIII-1 [17], the structural parameters of bellows should meet the requirements as shown in Figure 15.

**Figure 16:**
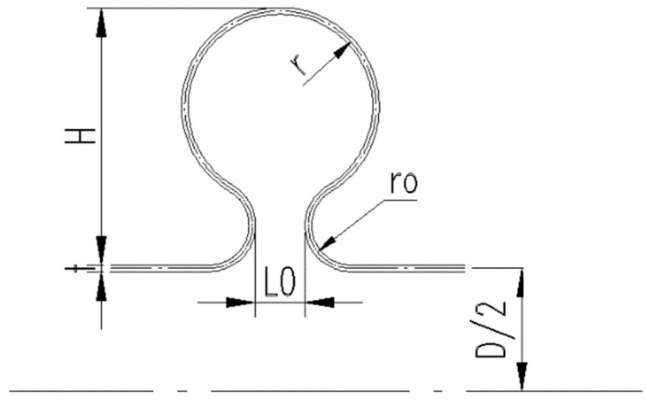
Dimensions of bellows in engineering practice.

Factors affecting the forming quality include pressure (P_1_) at the bulge stage, pressure(P_2_) at the fold stage, middle radius of small arc (r0), middle radius of big arc (r), middle diameter of tube blank (D), opening size of toroid (L0), wave height (H) and wall thickness (t). Dimensions of bellows are shown in Figure 16.

(1) $$P_{1}=\gamma_{1}\times \frac{2ntR_{m}}{D_{b}}$$

(2) $$P_{2}=\gamma_{2}\times \frac{2ntR_{m}}{D_{b}}$$

where, γ_1_: pressure coefficient bulge stage, $\frac{R_{eL}}{R_{m}}<\gamma_{1}<1$; γ_2_: pressure coefficient at fold stage, $\gamma_{2}\geq 1$; R_m_: tensile strength of tube blank at ordinary temperature; R_eL_: yield strength of tube blank at ordinary temperature; D_b_: inner diameter of tube blank; P_1_: pressureat the bulge stage; P_2_: pressureat the fold stage.

Usually, γ_1_ = 0.7–0.9, γ_2_ = 1–4. If γ_1_ and γ_2_ are too small, the ripple transition will not smooth enough. On the other hand, the wrinkling or even bursting defects could be induced by too large pressures.

In the design of toroidal bellows, the radius of small arc (r0) and the radius of large arc(r) are important structural factors. A large r0 will lead the wave-form of the toroidal bellows approach U-shape and the advantage of the large arc radius r will be weakened. Therefore, r0/r should be in a reasonable range and in this paper, r0/r is recommended to be in the range of 0.3–0.5 [18].

In bellows standards [19,20], ratio of opening size of toroid (L0) to large arc radius (r) is specified and the value should be less than 0.5, so in this paper, the dimensionless parameter L0/r is proposed to be 0.3–0.5 for studying its influence on the hydroforming quality of the bellows. In addition, wave height of bellows is mainly related to the diameter D of tube blank. Based on standards, r/D is usually to be 0.0108–0.08. For wide application in engineering, the tube blank diameter D is chosen from 300 mm to 2800 mm in this study.

### 3.2. Analysis of the Factors Influencing the Roundness of the Wave-Shape

Theoretical analysis and engineering experience tells that t/r0, r/D, r0/r, γ_2_, γ_1_, L0/r should have effects on the wave-shape quality. In addition, interactions of these factors may also have effects. For ensuring accuracy but not making the computation too complicated, the first level interaction between any two factors is considered. Orthogonal test is a mathematical tool to find importance order of factors. As the factors influencing the quality of bellows, orthogonal test is applied to figure out major factors [21]. In order to find interaction effects, the orthogonal test of L4(23) is executed which includes the interaction of the two considered factors. If the significance (denoted by R) of the “interaction” of any two factors is greater than that of the one or both factors, the interaction of these two factors will be taken into consideration [21]. Taking t/r0 and r/D as example, the results are listed in Table 6.

It is seen that R(a/2 h) of the interaction of t/r0 and r/D is larger than R(a/2 h) of r/D so the effect of the interaction of t/r0 and r/D on a/2 h should be taken into consideration. But R(δ) of the interaction of t/r0 and r/D is less than that of any of the two factors, so the effect of the interaction of t/r0 and r/D on δ can be ignored. This method is applied to study the interaction between other factors and it turns out that the influences of interactions of t/r0 and r/D, t/r0 and r0/r, t/r0 and γ_2_ on a/2 h should not be ignored and the effects of all interactions on δ can be neglected.

After numerous calculations, it is found that six factors, i.e., t/r0, r/D, r0/r, γ_2_, γ1, L0/r, and three interactions, i.e., t/r0 and r/D, t/r0 and r0/r, t/r0 and γ_2_ should be taken into consideration for their effects on the roundness of the bellows. In order to find the significance order of these factors, a much larger orthogonal experiment with nine factors and three levels was carried out regarding their effects on the roundness of the bellows [21].

**Figure 17:**
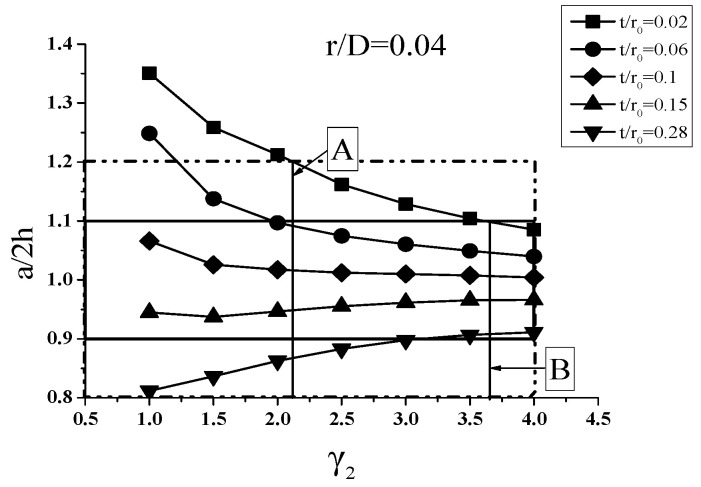
The relationship between a/2 h and γ_2_ for r/D = 0.04.

It turns out from the orthogonal experiment analysis that the most significant factors influencing the roundness of wave-shape are t/r0, r/D and γ_2_. If neglecting effects of other factors, it is easy to draw the relationship graphs between a/2 h and γ_2_ for given r/D as shown in Figure 17 which is based on the hydroforming simulation results when r/D = 0.04 as an example.

It is seen from Figure 17 that increasing γ_2_, i.e., increasing the pressure at the fold stage, a/2 h is getting close to 1. But if the pressure is too small, a/2 h may be too small or too large, depending on t/r0. If the a/2 h is too small, the shape will be close to the U-shape bellows. On the other hand, if the a/2 h is too large, the shape of the bellows will be close to the cylinder shape. In addition, it should be mentioned that increasing the pressure will reduce wall thickness and increase the difficulties of hydroforming.

In Figure 17, Line A is the line for γ_2_ on whose right a/2 h = 0.8–1.2 which meets the structural requirements of the bellows based on ASME VIII-1; Line B is the line for γ_2_ on whose right a/2 h = 0.9–1.1 which gives a more satisfactory shape of the bellows. But if γ_2_ is on the left of Line A, the shape of bellows could not meet the structural requirement. Specifically, for facilitating the engineering application, ranges of pressure coefficient γ_2_ at the fold stage for different r/D and different t/r0 are listed in Table 7 for a/2 h = 0.8–1.2 and Table 8 for a/2 h = 0.9–1.1.

### 3.3. Analysis of the Factors Influencing the Wall Thickness Reduction

It is found that L0/r, r/D, r0/r, t/r0, γ_1_, γ_2_ have effects on the relative wall thickness reduction δ and the interaction of factors can be ignored. Results after performing orthogonal tests with these factors find that the main factors influencing the relative wall thickness reduction are r/D, γ_1_, and γ_2_. For facilitating the engineering application, a formula for the relative wall thickness reduction δ is fitted based on the sufficient numerical simulations of the hydroforming process. It turns out that the relationship between δ and r/D, γ_1_ and γ_2_ is approximately linear. The user-defined module of Origin 9.1 [22] is used to fit the formula of δ, and the final fitted formula is:

(3) $$\begin{array}{l} \delta =2.0865(\frac{r}{D})+0.026\gamma_{1}-0.0092\gamma_{2}-0.1459\gamma_{1}\left(\frac{r}{D}\right)+0.4601\gamma_{2}\left(\frac{r}{D}\right)\\ \begin{array}{ll} where,\; & \frac{r}{D}\in \left[0.02,0.08\right]\\ & D\;\in \;\left[300,2800\right](mm)\\ & \gamma_{1}\in \left[0.7,0.9\right]\\ & \gamma_{2}\in \left[1,4\right] \end{array} \end{array}$$

The goodness of the fit of this formula is R^2^ = 0.9945, meaning the fitting is high. In addition, validations are also carried out to confirm the accuracy of fitting formula by comparing the numerical simulations and formula evaluations. The results are listed in Table 9. It is seen that the relative errors between simulation results and the formula calculation results are less than 7%, and the absolute errors are less than 2%, implying the formula is accurate enough for the engineering application.

It should be pointed out that hydroforming process is very complicated with many factors affecting the quality of the bellows. Expert system involving simulation results and engineering experiences is very helpful to control the parameters of the process and should be studied in the future.

## 4. Conclusions

Finite element simulations of hydroforming process of a monolayer and single-wave toroidal bellows and a two-layer and four-wave toroidal bellows were carried out and effects of the structural and operating parameters on the roundness of the wave-shape and the relative wall thickness reduction of bellows in the hydroforming process are analyzed. Quality control analysis is conducted and conditions for a better quality of the bellows are investigated. Conclusions are drawn as follows:

(1) Stress distributions at the bellows before and after unloading were examined. Large von Mises equivalent stress before unloading is largely distributed at the large arc before unloading but located at the transition between the large arc and the small arc with the value reduced by about 30% after unloading.
(2) The wall thickness reduction during the hydroforming process of bellows was investigated. During the hydroforming process of bellows, the relative wall thickness reduction at the wave crest could be as high as 18%.
(3) Compared with the practical measurements, the relative deviations of simulated wall thickness reduction and wave height are less than 3.78%, meaning that the finite element method is feasible and dependable for the numerical simulations of the hydroforming of the toroidal bellows.
(4) Factors t/r0, r/D and γ_2_ have the most significant influences on the roundness of the wave-shape. A better quality of the bellows requires reasonable value match of these factors.
(5) From the shape roundness consideration, γ_2_ = 1.7–3 is the best for a/2 h = 0.8–1.2 when t/r0 = 0.02–0.28 and r/D = 0.02–0.08. γ_2_ = 2.4–4 is the best for a/2 h = 0.9–1.1 when t/r0 = 0.02–0.28 and r/D = 0.04–0.08. These results are useful for selecting parameters in engineering design and manufacturing of bellows.
(6) A formula to compute the relative wall thickness reduction is fitted based on the sufficient numerical simulations of the hydroforming process and the numerical verification show that the formula is accurate enough for the engineering design and manufacture of bellows.

## Figures and Tables

**Table 1:** Displacements at wave height before and after unloading.

	Displacement at Outer Wall of Tube (mm)		Displacement at Inner Wall of Tube (mm)	
Stage	Before unloading	After unloading	Before unloading	After unloading
Value	77.821	77.263	78.173	77.613

**Table 2:** Displacements at wave height before and after unloading.

	Displacement at Outer Wall of Outer Tube (mm)		Displacement at Inner Wall of Outer Tube (mm)		Displacement at Outer Wall of Inner Tube (mm)		Displacement at Inner Wall of Inner Tube (mm)	
Stage	Before unloading	After unloading	Before unloading	After unloading	Before unloading	After unloading	Before unloading	After unloading
First wave	77.761	77.395	78.109	77.74	78.096	77.729	78.462	78.092
Second wave	77.878	77.494	78.22	77.836	78.22	77.835	78.581	78.194
Third wave	77.878	77.495	78.22	77.836	78.22	77.836	78.581	78.194
Fourth wave	77.761	77.395	78.109	77.74	78.096	77.729	78.462	78.092

**Table 3:** Wall thickness reduction of the bellows.

Outer/Inner Tube	Absolute Thickness Reduction of Outer Tube (mm)	Absolute Thickness Reduction of Inner Tube (mm)	Relative Thickness Reduction of Outer Tube	Relative Thickness Reduction of Inner Tube
First wave	0.345	0.363	17.25%	18.15%
Second wave	0.342	0.359	17.1%	17.95%
Third wave	0.341	0.358	17.05%	17.9%
Fourth wave	0.345	0.363	17.25%	18.15%

**Table 4:** Comparisons between simulation results and experimental results of the first wave.

Item	Simulations	Measurements	Relative Error *
Thickness of outer tube(mm)	1.655	1.72	3.78%
Thickness of inner tube (mm)	1.637	1.7	3.7%
Displacementsin wave height direction of outer tube (mm)	77.395	77.2	0.25%
Displacement in wave height direction of inner tube (mm)	78.092	77.3	1.02%

* $\mathrm{Relative}\;\mathrm{error}=\frac{\left|\mathrm{simulations}-\mathrm{measurements}\right|}{\mathrm{measurements}}\times 100\%$ font mix

**Table 5:** Comparison between simulation results and experimental results of the other waves.

Item	Simulations	Measurements	Relative Error
Displacements in wave height direction of the second wave (mm)	78.194	77.5	0.895%
Displacements in wave height direction of the third wave (mm)	78.194	77.5	0.895%
Displacements in wave height direction of the fourth wave (mm)	78.092	77	1.42%

**Table 6:** Orthogonal tests for studying the interaction between t/r0 and r/D.

Item	t/r0	r/D	Interaction	a/2 h	δ
1	0.02	0.02	1	1.1085	0.06
2	0.02	0.08	2	1.0831	0.19
3	0.28	0.02	2	0.7771	0.0665
4	0.28	0.08	1	0.9627	0.1953
R(a/2 h)	0.226	0.080	0.106		
R(δ)	0.006	0.129	0.001		

**Table 7:** Ranges of pressure coefficient at the fold stage for a/2 h = 0.8–1.2.

**r/D**	0.02	0.03	0.04	0.05	0.06	0.07	0.08
**t/r0**	0.02–0.28	0.02–0.28	0.02–0.28	0.02–0.28	0.02–0.28	0.02–0.28	0.02–0.28
**γ_2_**	>3	>2.3	>2.1	>2.1	>1.95	>1.9	>1.7

**Table 8:** Ranges of pressure coefficient at the fold stage for a/2 h = 0.9–1.1.

**r/D**	0.02 0.03		0.04	0.05	0.06	0.07	0.08
**t/r0**	>0.15	≤0.15	0.02–0.28	0.02–0.28	0.02–0.28	0.02–0.28	0.02–0.28
**γ_2_**	>4	=4	>3.65	>3.35	>3	>2.7	>2.4

**Table 9:** Accuracy test of formula for the relative wall thickness reduction.

Test No.	δ’	δ	r/D	γ_1_	γ_2_	D (mm)	Absolute Error	Relative Error
1	6.40%	5.96%	0.02	0.75	2.5	1000	0.44%	6.95%
2	9.40%	9.16%	0.028	0.89	3.7	1256	0.24%	2.60%
3	11.00%	10.56%	0.032	0.86	3.7	2500	0.44%	4.05%
4	12.60%	12.50%	0.038	0.86	3.4	800	0.10%	0.76%
5	12.00%	11.34%	0.04	0.8	1.5	2000	0.66%	5.5%
6	16.50%	15.97%	0.048	0.83	3.4	2500	0.53%	3.19%
7	15.05%	15.24%	0.05	0.7	2.5	533	0.19%	1.29%
9	19.00%	18.45%	0.058	0.83	2.8	2000	0.55%	2.87%
10	18%	17.58%	0.06	0.8	2	830	0.42%	2.33%
11	22.00%	23.01%	0.066	0.86	3.7	2000	1.01%	4.59%
12	22.00%	21.14%	0.068	0.89	2.5	1256	0.86%	3.90%
14	19.00%	18.17%	0.07	0.8	1	480	0.83%	4.37%
15	27.00%	26.63%	0.076	0.83	3.7	1256	0.37%	1.37%
16	28.00%	28.12%	0.078	0.8	4	800	0.12%	0.43%
17	28.50%	27.31%	0.08	0.86	3.4	2500	1.19%	4.17%

δ’: Relative wall thickness reduction from numerical simulations; δ: Relative wall thickness reduction from fitting formula; Absolute error = |δ′-δ|; Relative error = $\frac{\left|\delta \prime -\delta \right|}{\delta \prime }$

## Data Availability

The data presented in this study are available on request from the corresponding author. The data are not publicly available.
